# Systematic Characterization of *Cacopsylla chinensis* as a Potential Vector of *Erwinia amylovora* on Korla Fragrant Pear in Xinjiang, China

**DOI:** 10.3390/insects17050487

**Published:** 2026-05-09

**Authors:** Yulin Yuan, Zhe He, Luwei Wang, Xinlu Liu, Zhenya Liu, Yapeng Li, Huimin Liu, Wangbin Zhang

**Affiliations:** 1 National and Local Joint Engineering Laboratory of High Efficiency and Superior-Quality Cultivation and Fruit Deep Processing Technology of Characteristic Fruit Trees in South Xinjiang, Alaer 843300, China; yuanyulin2026@163.com (Y.Y.); zhehe@taru.edu.cn (Z.H.); wangluwei202605@163.com (L.W.); lxldzl@126.com (X.L.); nxylzy@taru.edu.cn (Z.L.); liyapengtada@163.com (Y.L.); lhuimin@163.com (H.L.); 2 Key Laboratory of Integrated Pest Management of Xinjiang Production and Construction Corps in Southern Xinjiang, Alaer 843300, China; 3 Scientific Observation and Experiment Station for Crop Pests (Alaer), Ministry of Agriculture and Rural Affairs, Alaer 843300, China; 4 College of Agriculture, Tarim University, Alaer 843300, China

**Keywords:** *Erwinia amylovora*, *Cacopsylla chinensis*, synergistic damage, pathogenicity, vector-borne carriage

## Abstract

Pear fire blight, caused by the bacterium *Erwinia amylovora*, is a destructive disease affecting Rosaceae plants. Although insect transmission is well-documented, most studies have focused on pollinators, with limited attention to psyllids. Chinese pear psyllid (*Cacopsylla chinensis*) is a major piercing–sucking pest of pear trees, yet its role in the transmission of *E. amylovora* remains unclear. Here, we investigated the distribution of *E. amylovora* in and on *C. chinensis* and the synergistic damage (i.e., *C. chinensis* creates invasion wounds and nutrient-rich niches for *E. amylovora* via piercing–sucking feeding, while the pathogen enhances the vector’s fitness to promote disease spread). Field and laboratory assays confirmed severe synergistic symptoms. *E. amylovora* was isolated from all life stages and body parts of *C. chinensis*, with significantly higher pathogen loads and virulence in internally harbored strains compared to external ones. Specifically, *E. amylovora* loads in nymphs were significantly higher than those in adults, and strains from the digestive system and female reproductive organs caused a 3- to 9-fold higher disease index on pear leaves at 7 days post-inoculation compared to body surface isolates. This study provides evidence that *C. chinensis* acts as a crucial vector for *E. amylovora* in Xinjiang, laying a theoretical basis for the precise integrated management of this pest–disease complex.

## Simple Summary

Pear fire blight is a destructive bacterial disease that harms pear trees and related fruit crops, causing severe economic losses for fruit growers. While this disease is known to spread via insects, most past research has only focused on pollinators like honeybees, with little attention paid to the Chinese pear psyllid—the most common insect pest of pear trees. It has long been unclear whether this psyllid can spread the bacteria that cause pear fire blight. In this study, we investigated how the psyllid carries the disease-causing bacteria and how the insect and bacteria together damage pear trees. We found that the psyllid can carry the bacteria across all its life stages and in all body parts; bacteria inside the insect are significantly higher in virulence (*p* < 0.05), especially those in the digestive system and female reproductive organs. This study provides evidence that the Chinese pear psyllid is a key carrier of pear fire blight bacteria in Xinjiang, China. Our findings provide a scientific basis for precise pest and disease control, helping protect pear crops and secure growers’ livelihoods.

## 1. Introduction

The Korla pear (*Pyrus sinkiangensis* Yü) is a characteristic fruit tree in Xinjiang, China, and possesses significant economic value [1]. As one of the major diseases affecting Korla pear, pear fire blight poses a serious threat to the development of the local pear industry [2]. *E. amylovora*, the causal agent of pear fire blight, can infect flowers, leaves, branches, and trunks of Rosaceae plants, resulting in a black and scorched appearance of infected tissues that resembles fire damage [3]. Furthermore, this pathogen can cause fruit malformation or rot, significantly reducing the yield and quality of Korla pears [4]. *E. amylovora* has multiple transmission pathways, including natural factors (wind, rain, and insect vectors) and human activities [4,5]. Notably, mechanical wounds on plant surfaces caused by insect feeding significantly increase the pathogen’s infection probability [6,7,8,9].

The invasion of insects has a significant impact on the outbreak of plant diseases [10,11,12]. Plant pathogens have evolved diverse transmission strategies, including utilizing insects as vectors to survive adverse environments [13]. During the feeding process of insects on infected plants, numerous plant pathogens invade the insect body and persist using the insect as a host [14]. This process usually induces morphological, physiological, or behavioral changes in insects, thereby facilitating the growth and reproduction of the pathogen [15]. Many plant pathogens can be carried and survive in insects; for example, aphids and psyllids are capable of carrying pathogens through circulative transmission and transmitting them to healthy host plants during feeding [11,16,17].

Pathogenicity of insect-vectored plant pathogens is a critical factor governing the epidemiology of plant diseases [18]. However, not all pathogens that colonize insects retain the ability to cause disease in their host plants. For example, *Candidatus* Liberibacter asiaticus (the causal agent of citrus Huanglongbing) can stably persist in multiple internal and external tissues of mealybugs, yet it loses its pathogenicity and cannot incite disease in host plants [19]. Previous studies have demonstrated that *E. amylovora* can be carried by various insect species and retains pathogenicity. For example, honeybees are capable of mechanically carrying *E. amylovora* on their body surfaces and transmitting the pathogen to induce plant infections [20]; *E. amylovora* can also survive both internally and externally in the Mediterranean fruit fly (*Ceratitis capitata*) while maintaining pathogenicity [21]; and *Drosophila melanogaster* can acquire pathogenic *E. amylovora* from sludge under diverse biological environments through feeding, and subsequently transmit the pathogen [22].

*C. chinensis* is a dominant pest in Korla fragrant pear orchards of Xinjiang and has been confirmed to carry *E. amylovora* [23]. However, the potential effects of different carriage modes (external adhesion and internal colonization) on the biological characteristics and subsequent pathogenicity of the pathogen remain poorly understood. Most existing studies have focused on the impacts of *E. amylovora* on its insect vectors, while largely overlooking the adaptive changes that the pathogen may undergo within this unique insect-associated ecological niche. Therefore, this study aimed to: (1) clarify the pathogen-carrying capacity of *C. chinensis* across different life stages (nymph vs. adult) and carriage modes (internal colonization vs. external adhesion); (2) compare the pathogenicity differences in *E. amylovora* strains isolated from different tissues and organs of *C. chinensis*; and (3) reveal the synergistic damage mechanism between *C. chinensis* and *E. amylovora* on Korla fragrant pear. This work is of great significance for elucidating the transmission mechanism of pear fire blight and formulating targeted prevention and control strategies. Specifically, we proposed three testable hypotheses: (1) *E. amylovora* can colonize both internal tissues and external surfaces of *C. chinensis* across all life stages; (2) internally colonized *E. amylovora* exhibits higher proliferation capacity and virulence than externally adhered strains; and (3) *E. amylovora* strains from different organs of *C. chinensis* show differences in pathogenicity due to tissue-specific microenvironmental selection.

## 2. Materials and Methods

### 2.1. Sample Collection and Processing

To evaluate the ability of *C. chinensis* to carry *E. amylovora*, field surveys and sample collections were conducted in the Korla pear orchard affiliated with Tarim University (40°3′29′′ N, 81°17′32′′ E) from June to September 2024. Adult *C. chinensis* resting on leaves were captured by inverting 1.5 mL centrifuge tubes over them, while leaves with *C. chinensis* nymphs and suspected pear fire blight symptoms were collected using self-sealing bags. After collection, all samples were subjected to qualitative detection of *E. amylovora* via both PCR amplification and bacterial isolation and culture. Additionally, the collected leaves were incubated in an artificial climate chamber (GXZ-436B, Ningbo Kesheng experimental instrument Co., Ltd., Ningbo, China) at 28 °C with a relative humidity of 65% ± 5%.

### 2.2. Insects and Rearing Conditions

*E. amylovora*-carrying *C. chinensis* individuals were collected from the Korla pear orchard of Tarim University, where the host plants were 15–20-year-old *E. amylovora*-infected Korla pear trees. In contrast, *E. amylovora*-free *C. chinensis* was collected from pear orchards in Xianyang City, Shaanxi Province, a non-endemic area of *E. amylovora*. All samples were confirmed to be free of *E. amylovora* via species-specific PCR detection. (34°32′39′′ N, 107°17′10′′ E). The *E. amylovora*-carrying and -free *C. chinensis* were separately inoculated onto healthy Korla pear trees enclosed in mesh cages. All insects were reared in an insectary under controlled conditions: a photoperiod of 12 h light:12 h dark (12L:12D), a temperature of 25 °C ± 1 °C, and a relative humidity (RH) of 65% ± 5%. Continuous monitoring of temperature and humidity was conducted with a hygrothermograph (LYWSD02MMC, Beijing Xiaomi Technology Co., Ltd., Beijing, China).

### 2.3. Extraction of E. amylovora DNA and PCR Amplification

Collected adult and nymph *C. chinensis* individuals were rinsed with sterile water separately, followed by genomic DNA extraction using a bacterial genomic DNA extraction kit (Tiangen Biotech, Beijing, China). The extracted DNA was stored at −20 °C until use. Genomic DNA amplification was performed using the specific primers RS24580-205F/R [24] following the optimized PCR program: pre-denaturation at 95 °C for 5 min; 35 cycles of denaturation at 95 °C for 30 s, annealing at 60 °C for 30 s, and extension at 72 °C for 30 s; and a final extension at 72 °C for 10 min. For PCR identification of *E. amylovora* strains, six strains (Y-1, RN, RW, CN, CW, ML) isolated from the internal/external surfaces of *C. chinensis* and pear leaves, along with one positive control strain (LHY), were used (total of 7 strains). Sterile water was used as the negative control, and the confirmed pathogenic *E. amylovora* strain LHY was used as the positive control in all PCR amplifications. Each PCR reaction was performed with three technical replicates to ensure the reliability of results. Amplification with the specific primers RS24580-205F/R [24] yielded a target gene fragment of approximately 205 bp, confirming that these strains belong to the European race of *E. amylovora*. All PCR amplifications were performed with three biological replicates and three technical replicates for each sample. To prevent contamination, all PCR reagents were prepared in a sterile biosafety cabinet, and dedicated pipettes with filter tips were used for reagent preparation and template addition. No template control (NTC) reactions were included in every PCR run to monitor for potential reagent contamination, and no target bands were observed in any NTC reactions throughout the study. Detailed information on the strains is provided in Supplementary Table S1.

### 2.4. Isolation of E. amylovora Carried by C. chinensis

Bacterial isolation was performed with modifications based on Mollaei SN et al. [25]. The major optimizations included: (1) surface disinfection of insect samples in 0.5% sodium hypochlorite was shortened from 30 s to 5 s to preserve viable internal bacteria; (2) triple sterile water rinses were added post-disinfection, and the final rinse was plated on LB medium to validate complete surface sterilization; (3) serial dilutions were expanded to 10^−1^–10^−7^ for improved single-colony separation. Samples were sourced from Korla fragrant pear orchards affected by pear fire blight. *E. amylovora* was isolated from the external surfaces and internal tissues of *C. chinensis* (adults and nymphs) and diseased pear leaves. In brief, washed *C. chinensis* adults and nymphs were disinfected in 0.5% sodium hypochlorite for 5 s and rinsed thrice with sterile water, then ground into a uniform homogenate. Five types of samples were prepared for bacterial isolation, including two internal tissue samples (adult homogenate, nymph homogenate), two external surface samples (insect washing solution, soaking solution of field-collected leaves with synergistic disease symptoms), and one honeydew sample (nymph-secreted honeydew) were serially diluted 10-fold in PBS to 10^−1^–10^−7^. Each dilution was spread onto LB solid medium, and plates were incubated at 28 °C in a constant-temperature incubator.

### 2.5. Comparison of CFU Counts of E. amylovora Strains from Different Sources

To quantify the abundance of *E. amylovora* carried internally and externally by *C. chinensis*, purified strains from different sources were separately cultured in LB liquid medium with shaking for 12 h. The OD_600_ value of each bacterial suspension was uniformly adjusted to 1.0 with sterile water to prepare the stock solution. For ease of pipetting, the stock solution was transferred to 2 mL sterile centrifuge tubes using a pipette prior to dilution. Subsequently, nine new 2 mL sterile centrifuge tubes were numbered sequentially, and 900 μL of sterile PBS buffer was added to each tube. A 100 μL aliquot of the stock solution was pipetted into one of the tubes, resulting in 1 mL of bacterial suspension. The suspension was thoroughly mixed by vortexing to prepare the first dilution gradient (10^−1^). Then, 100 μL of the 10^−1^ gradient suspension was serially diluted to obtain final dilutions of 10^−5^, 10^−6^, and 10^−7^. This serial dilution protocol was applied to all strains from different sources. A 100 μL aliquot of each diluted suspension was uniformly spread onto the surface of NSA medium (Nutrient Sucrose Agar, PM 7/20 (3) *Erwinia amylovora*; EPPO Bulletin) using a sterile spreader. Each dilution gradient was spread in three biological replicates. The inoculated Petri dishes were incubated in a bacterial incubator at 28 °C with a relative humidity of 65% ± 5%. After 48 h of incubation, colony-forming unit (CFU) counting was performed for the three biological replicates of each dilution gradient. Counting was conducted manually by two independent researchers in a double-blind manner, and plates with 30–300 colonies were used for final CFU calculation. The average count of the two researchers was used for subsequent statistical analysis. Data were subjected to one-way analysis of variance (ANOVA) followed by Tukey’s HSD test for multiple comparisons, using IBM SPSS Statistics 20.0. Differences were considered significant at *p* < 0.05.

### 2.6. Comparison of Pathogenicity of E. amylovora Strains Carried by C. chinensis

To simulate the natural growth environment of *E. amylovora* strains carried internally and externally by *C. chinensis*, 1 mL aliquots of each sample prepared in Section 2.5 (adult homogenate, nymph homogenate, adult/nymph rinse solution, nymph-secreted honeydew, and soaking solution of field-collected leaves with synergistic disease symptoms) were inoculated into LB liquid medium for shaking culture. After 12 h of incubation, 10 mL of each bacterial suspension was aspirated and centrifuged at 5000 rpm for 5 min. The supernatant was discarded, and the pellet was washed three times with sterile PBS buffer, then resuspended in PBS buffer to adjust the OD_600_ value to 1.0. Tender, healthy branches, leaves, and fruits of Korla fragrant pear were collected. A 100 μL aliquot of each bacterial suspension (OD_600_ = 1.0) from different sources was inoculated into the corresponding pear tissues using a 1 mL syringe. Each strain was tested in three independent biological groups, with 10 biological replicates (independent pear tissue samples) per group. All inoculated pear tissues were randomly assigned to different treatment groups using a random number table, and the incubation position of each sample in the artificial climate chamber was randomized daily to avoid position effects. The inoculated leaves, branches, and young fruits were incubated in an artificial climate chamber at 28 °C with 65% ± 5% relative humidity. After inoculation, the disease index of branches, leaves, and fruits was recorded every 24 h. The disease index was evaluated based on a modified method from Paprstein et al. [26], and the detailed modified criteria are provided in Supplementary Table S2. Disease index and incidence data were analyzed using repeated-measures ANOVA, with Tukey’s HSD test for multiple comparisons at each time point (*p* < 0.05), as detailed in Section 2.8 (Statistical Analysis).

### 2.7. Construction and Analysis of Phylogenetic Tree

The amplified PCR products were sent to Shanghai Sangon Biotech Co., Ltd. (Shanghai, China) for Sanger sequencing. The obtained sequences were submitted to the GenBank database for sequence alignment, and sequences of closely related species were downloaded from the same database. Phylogenetic analysis was performed via the Neighbor-Joining (NJ) in MEGA 11, with 1000 bootstrap replicates for assessing nodal support. Accession numbers for each sequenced sequence were applied for in GenBank. Detailed information of the sequences involved in the phylogenetic analysis is provided in Supplementary Table S3.

### 2.8. Statistical Analysis

All statistical analyses were performed using SPSS 20.0 software (IBM Corp., Armonk, NY, USA), and graphs were plotted using GraphPad Prism 9.0 software (GraphPad Software, San Diego, CA, USA). For CFU count data, one-way ANOVA was used to compare differences among groups, followed by Tukey’s HSD post hoc test for multiple pairwise comparisons, with a significance threshold set at *p* < 0.05. For disease index and incidence data collected at multiple time points, repeated-measures ANOVA was performed. Tukey’s HSD test was used for multiple comparisons among groups at each individual time point (*p* < 0.05). For phylogenetic analysis, bootstrap values were calculated based on 1000 replications to evaluate the robustness of tree nodes. Throughout the study, biological replicates were defined as independent experiments performed using different batches of insects and plant materials, while technical replicates were defined as repeated measurements of the same sample. All experiments included at least three independent biological replicates, and specific replicate numbers for each assay are detailed in the corresponding method sections. The sample size for each experiment was determined based on previous studies on insect vector–pathogen interactions [23] to ensure sufficient statistical power. All positive and negative controls used in each experiment are clearly described in the corresponding method sections, and all control treatments were included in every experimental run to validate the reliability of the assay system.

## 3. Results

### 3.1. Observation and Verification of Field Synergistic Damage Symptoms Caused by C. chinensis and E. amylovora

**Figure 1:**
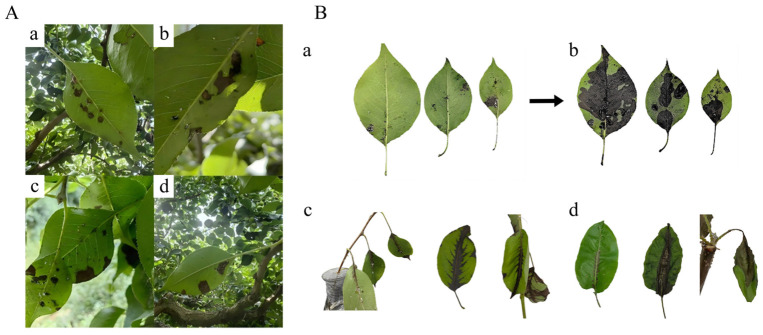
Synergistic damage symptoms of Korla pear by *C. chinensis* and *E. amylovora*. (**A**) Field symptoms: leaf damage, honeydew secretion, and fire blight-like lesions. (**B**) Indoor verification: (**a**) Asymptomatic field leaves; (**b**) leaves after humidified incubation; (**c**) leaves co-infested with *C. chinensis* and *E. amylovora*; (**d**) leaves infested only by sterile *C. chinensis*. A total of 180 asymptomatic infested leaves were collected from 9 pear trees for the indoor assay.

In Korla pear orchards with pear fire blight outbreaks, a large number of leaves infested by *C. chinensis* were observed to exhibit symptoms similar to those of pear fire blight (Figure 1A). Randomly distributed necrotic black lesions appeared on these leaves; under favorable climatic conditions, these lesions continuously expanded and eventually coalesced, leading to extensive necrosis of leaf tissue. Leaves infested by *C. chinensis* with feeding spots and no visible necrotic lesions or vein blackening were collected (Figure 1B(a)). A field survey of 180 infested leaves from 9 pear trees showed that 43.3% of these asymptomatic leaves developed typical fire blight symptoms after 7 days of indoor moist incubation (Figure 1B(b)). Nymphs of *C. chinensis* either carrying or not carrying *E. amylovora* (*n* = 5) were separately placed on healthy Korla pear leaves (*n* = 90). After 7 days of observation, leaves fed on by *E. amylovora*-carrying *C. chinensis* nymphs showed blackening of leaf veins, which gradually spread to the surrounding tissues, forming symptoms consistent with the characteristic signs of pear fire blight. During this period, the pear leaves remained turgid without wilting (Figure 1B(c)). In contrast, leaves fed on by *E. amylovora*-free *C. chinensis* nymphs only developed black spots on both sides of the leaf veins without spreading, and the leaf veins remained normal in color (no blackening). With prolonged infestation by *C. chinensis* nymphs, the pear leaves gradually wilted (Figure 1B(d)).

### 3.2. Detection, Isolation, and Pathogenicity of E. amylovora from Internal and External Surfaces of C. chinensis to Korla Pear

**Figure 2:**
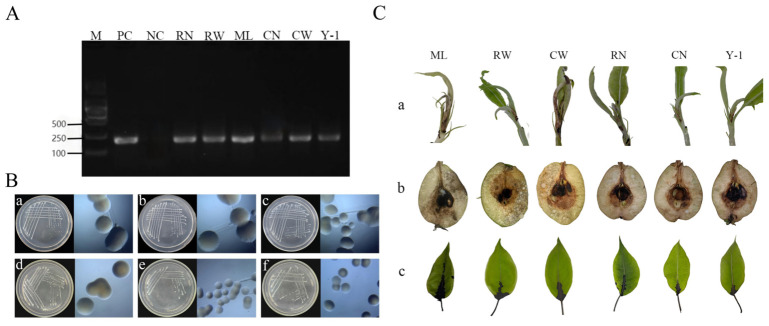
Detection, colony isolation and pathogenicity of *E. amylovora* carried by *C. chinensis*. (**A**) Detection of *E. amylovora* in *C. chinensis* and pear leaves. (**B**) Colonies of *E. amylovora* from internal and external parts of *C. chinensis*. Panels (**a**–**f**) correspond to isolates from ML, RW, CW, RN, CN, and Y-1, respectively. (**C**) Disease symptoms on pear tissues inoculated with corresponding *E. amylovora* strains: (**a**) pear shoots, (**b**) young fruits, and (**c**) young leaves.

*E. amylovora* strains were isolated from six sources: the external surface of adult *C. chinensis* (CW), internal tissues of adult *C. chinensis* (CN), nymph-secreted honeydew (ML), field-collected leaves with synergistic damage (Y-1), internal tissues of nymphs (RN), and external surface of nymphs (RW). A known pathogenic *E. amylovora* strain was used as the positive control (PC), and sterile water served as the negative control (NC). PCR detection was performed using the specific primers RS24580-205F/205R to target *E. amylovora* in samples from the internal/external surfaces of *C. chinensis*, honeydew, and synergistically damaged leaves (Figure 2A). Each sample contained 10 individuals of *C. chinensis*. Three sample groups were collected, with three independent biological replicates for PCR detection. The results showed that the detection frequency of *E. amylovora* was 100% in internal tissues of adults (CN) and nymphs (RN), 77.8% in adult external surfaces (CW), 66.7% in nymph external surfaces (RW), 88.9% in nymph honeydew (ML), and 100% in field-infected leaves (Y-1), confirming that *E. amylovora* was widely present in the internal and external surfaces of *C. chinensis* as well as in the leaves infested by the insect. All isolated *E. amylovora* strains from different sources (internal/external insect surfaces and infested leaves) exhibited consistent colony morphology on LB solid medium: milky white, single colonies with a raised center, viscous texture, hemispherical shape, and neat edges. No apparent morphological differences were observed among the strains (Figure 2B). All *E. amylovora* strains from different sources were pathogenic to Korla pear. Specifically, black necrotic lesions appeared rapidly at the petiole inoculation sites, accompanied by bacterial ooze exudation, and the lesions spread rapidly along the petioles and leaf veins to the leaf blades. Abundant white bacterial ooze exuded from the surface of young fruits. The inoculation sites on tender branches turned black, and reddish-brown bacterial ooze was observed on the young leaves at the shoot tips (Figure 2C).

### 3.3. Differences in CFU Counts and Pathogenicity of E. amylovora Strains Carried Internally and Externally by C. chinensis

**Figure 3:**
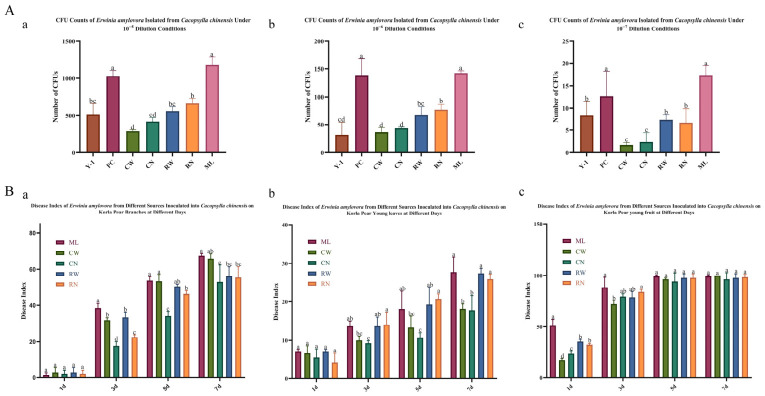
CFU quantification and pathogenicity of *E. amylovora* carried by *C. chinensis*. (**A**) CFU counts of *E. amylovora* isolated from different developmental stages and body niches of *C. chinensis* under three dilution conditions: (**a**) 10^−5^ dilution, (**b**) 10^−6^ dilution, and (**c**) 10^−7^ dilution. (**B**) Disease indices of Korla pear tissues inoculated with different *E. amylovora* strains over 7 days post-inoculation (dpi): (**a**) pear branches, (**b**) young leaves, and (**c**) young fruits. Data are mean ± SD (*n* = 3). Different letters indicate significant differences (*p* < 0.05) by Tukey’s HSD test (SPSS 20.0).

*E. amylovora* isolates acquired from different developmental stages (adults and nymphs) and body niches (internal tissues vs. external surfaces) of *C. chinensis* displayed varied colony abundances on NSA medium. As shown in Figure 3A, at dilutions of 10^−5^ to 10^−7^, CFU abundances of internally and externally carried isolates showed no statistical disparity within either adults or nymphs (Student’s *t*-test, *p* > 0.05). However, nymph-associated isolates presented markedly higher CFU loads than adult counterparts for both internal and external populations (one-way ANOVA, *p* < 0.05). Overall, isolates from honeydew (ML) yielded the greatest CFU values and exhibited pronounced disparities from most other groups, except the positive control (PC), whereas isolates from adult external surfaces (CW) recorded the lowest CFU levels. For example, at a dilution of 10^−6^, the CFU abundance of ML was 4.7-fold higher than that of CW (Figure 3A(b)). Disease indices on inoculated Korla pear branches exhibited notable temporal variations among *E. amylovora* isolates from different sources (Figure 3B(a)). At 3 days post-inoculation (dpi), honeydew-derived strains (ML) triggered substantially elevated disease indices relative to other isolates; this gap became indistinct between honeydew and adult external surface (CW) isolates at 5–7 dpi. From 3 to 5 dpi, isolates from adult internal tissues (CN) maintained consistently lower pathogenic levels than other groups, while nymph internal tissue (RN) isolates produced evidently higher disease indices than CN strains. On inoculated pear leaves, disease index patterns also showed apparent discrepancies across isolate sources over time (repeated-measures ANOVA, *p* < 0.05, Figure 3B(b)). At 3 dpi, isolates from honeydew (ML), nymph external surfaces (RW), and nymph internal tissues (RN) caused statistically higher disease indices than other strains (*p* < 0.05). From 3 to 7 dpi, RN isolates remained more virulent than CN isolates (*p* < 0.05), and ML isolates sustained high pathogenicity throughout the observation period. On inoculated pear fruits, disease indices displayed clear differences between nymph- and adult-derived isolates from 1 to 3 dpi (repeated-measures ANOVA, *p* < 0.05, Figure 3B(c)). At 1 dpi, honeydew (ML) isolates induced the most severe symptoms. RN isolates were statistically more pathogenic than CN isolates, and RW isolates showed greater virulence than CW isolates (*p* < 0.05). By 3 dpi, CW isolates produced markedly lower disease indices than ML and RN isolates (*p* < 0.05).

### 3.4. Bacteria-Carrying Status and Pathogenicity Differences in E.amylovora in Different Organs of Adult C. chinensis

**Figure 4:**
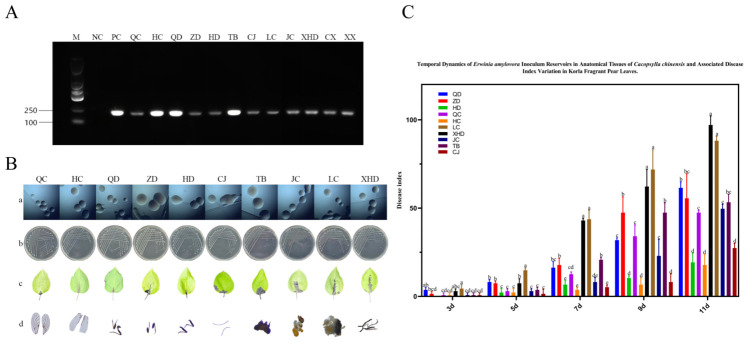
Detection and pathogenicity of *E. amylovora* in anatomical parts of adult *C. chinensis* on Korla fragrant pear leaves. (**A**) Detection of *E. amylovora* in anatomical parts of adult *C. chinensis*. (**B**) Disease symptoms, colonies and microscopic images of corresponding *E. amylovora* isolates: (**a**) microscopic morphology of the bacterial isolates, (**b**) colony morphology on LB medium, (**c**) disease symptoms on inoculated Korla fragrant pear leaves, and (**d**) the dissected anatomical tissues of *C. chinensis* used for bacterial isolation. (**C**) Disease indices of pear leaves inoculated with *E. amylovora* from different anatomical parts over time. Data are mean ± SD (*n* = 3). Different letters indicate significant differences (*p* < 0.05) by Tukey’s HSD test (SPSS 20.0).

To further characterize the *E. amylovora*-carrying status of organs in adult *C. chinensis*, we performed PCR detection on female and male adults, as well as dissected tissues, using species-specific primers (RS24580-205F/205R). Both sexes tested positive for *E. amylovora* (Figure 4A), and the bacterium was detected in all examined tissues, including the head, forewings, hindwings, forelegs, midlegs, hindlegs, digestive system, and male/female reproductive systems. Ten strains (QC, HC, QD, ZD, HD, TB, CJ, LC, JC, XHD) were isolated from these tissues: forewings (QC), hindwings (HC), forelegs (QD), midlegs (ZD), hindlegs (HD), head (TB), antennae (CJ), female reproductive system (LC), male reproductive system (JC), and digestive system (XHD). All isolates showed identical colony morphology on LB medium—milky white, viscous, hemispherical, with smooth edges and raised centers—and were indistinguishable from the positive control (PC; Figure 4B). All strains induced typical pear fire blight symptoms on tender Korla pear leaves, with significant differences in disease indices across strains at all observation time points (repeated-measures ANOVA, *p* < 0.05; Figure 4C). From 3 to 5 days post-inoculation (dpi), strain LC (female reproductive system) induced significantly higher disease indices than all other strains (*p* < 0.05), while no significant difference was observed between LC and strain XHD (digestive system) at 7 dpi (*p* > 0.05). Strains HD (hindlegs), HC (hindwings), and CJ (antennae) consistently caused the lowest disease indices across all time points (*p* < 0.05), whereas strains TB (head), QD (forelegs), and ZD (midlegs) maintained relatively high pathogenicity throughout the observation period. Strains isolated from the digestive system and female reproductive system resulted in 3- to 9-fold higher disease indices on pear leaves at 7 days post-inoculation, compared with strains from the body surface.

### 3.5. Analysis of Genetic Sequence Differences in E. amylovora Carried by C. chinensis

**Figure 5:**
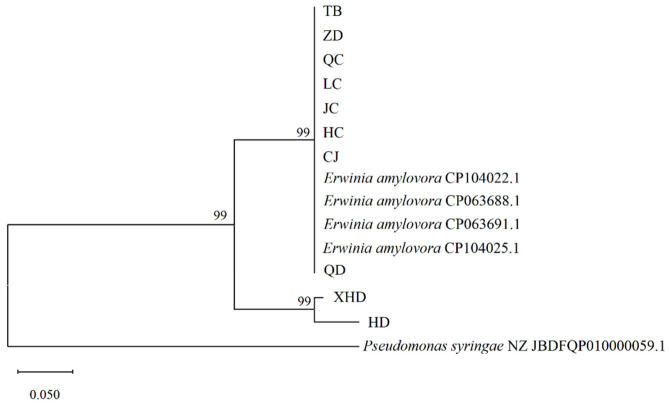
Phylogenetic tree of *E. amylovora* strains from different anatomical parts of adult *C. chinensis*. Bootstrap values (1000 replicates) are shown at the nodes; the scale bar indicates a genetic distance of 0.05. *Pseudomonas syringae* served as the outgroup.

The species-specific primer pair RS24580-205F/205R for *E. amylovora* was used to amplify sequences from strains isolated from different anatomical parts of adult *C. chinensis*, followed by phylogenetic analysis (Figure 5). Sequence alignment of the tested strains with *E. amylovora* reference sequences (CP063688.1, CP063691.1 from the US; CP104022.1, CP104025.1 from Xinjiang, China) in the NCBI database revealed 99% sequence identity between all tested strains and the references, confirming that all isolates belong to *E. amylovora*. A Neighbor-Joining (NJ) phylogenetic tree was constructed based on the aligned sequences, rooted with *Pseudomonas syringae* as the outgroup. The results indicated that all tested strains and *E. amylovora* reference strains formed a distinct monophyletic group with 99% bootstrap support, completely separated from the outgroup. Within this major clade, strains ZD, TB, QC, LC, JC, HC, CJ, and QD clustered in the same subclade as the reference isolates, while XHD and HD formed a separate but closely related subclade, both with 99% bootstrap support. Integrated analysis of colonial morphology, pathogenicity, and phylogenetic relationships confirmed that different body parts of adult *C. chinensis* can carry viable and pathogenic *E. amylovora*. Notably, the 99% sequence identity observed here confirms the isolates’ taxonomic identity as *E. amylovora* but does not indicate novel genetic variants, as high similarity alone does not support claims of novelty.

## 4. Discussion

The present study systematically characterized the carriage of *E. amylovora* across different developmental stages and anatomical tissues of *C. chinensis*, as well as the pathogenic variation in vector-borne strains. Our results directly address the predefined study objectives and hypotheses: we confirmed that *C. chinensis* can carry viable and pathogenic *E. amylovora* via both mechanical (external attachment) and biological (internal colonization) routes, and identified significant differences in the pathogenicity of strains from different sources (*p* < 0.05). These findings fill critical knowledge gaps in the interaction between *C. chinensis* and *E. amylovora*, and provide novel insights into the epidemic dynamics of pear fire blight.

Synergistic damage from pest-pathogen interactions drives substantial economic losses in global agriculture [27,28,29,30], as pests and pathogens often form mutually promoting, interdependent relationships that amplify disease epidemics [31,32]. Consistent with classic vector–pathogen systems (including psyllid-transmitted citrus Huanglongbing [33], leafhopper/aphid-borne phytopathogens [16], and thrips-transmitted viral diseases [34]), our field observations revealed that Korla pear leaves infested by *C. chinensis* developed fire blight-like symptoms, which was further validated by laboratory assays. We confirmed that *C. chinensis* and *E. amylovora* exert synergistic damage to pear hosts via a dual mechanism: piercing–sucking feeding creates mechanical wounds that facilitate pathogen invasion, while vector-secreted honeydew provides a nutrient-rich reservoir for *E. amylovora* colonization and proliferation.

The survival and pathogenicity of *E. amylovora* in insect vectors have been reported in other species, including flies [35] and stink bugs [36], but the carriage characteristics and pathogenic variation in *E. amylovora* in *C. chinensis* remained uncharacterized prior to this work. Here, we provide systematic evidence *E. amylovora* can colonize all tested anatomical tissues of adult *C. chinensis*, with viable strains isolated from both internal and external surfaces of the vector. We also detected viable *E. amylovora* across nymph and adult stages of *C. chinensis*, indicating a more intimate vector–pathogen interaction than previously recognized. Critically, overwintering adult *C. chinensis* may act as a key reservoir for *E. amylovora* during host-scarce non-growing seasons [37], which has important implications for understanding the overwintering and annual recurrence of pear fire blight in the field.

All *E. amylovora* isolates in this study showed consistent colony morphology on NA medium, but exhibited marked variation in pathogenicity across different host tissues (branches, leaves, fruits). This phenomenon is consistent with previous reports that phytopathogens modulate virulence factor expression and metabolic pathways to adapt to vector microenvironments [38,39], facilitating survival and transmission in *C. chinensis*. We found that strains from nymphs (both internal and external) generally had higher virulence than adult-derived strains. This may be explained by the longer feeding duration of nymphs on young tender tissues [40], higher pathogen acquisition efficiency [41,42], or the less developed immune system of nymphs compared with adults, which imposes weaker selection pressure on pathogen proliferation [43]. This could be attributed to several potential reasons, which remain to be further verified.

Notably, strains isolated from *C. chinensis* honeydew had the highest CFU counts and consistently strong pathogenicity across all assays. Honeydew secreted by *C. chinensis* nymphs is rich in sugars, amino acids, and other nutrients, acting as a natural medium for *E. amylovora* growth [44], which likely drives the high pathogen load and strong virulence of honeydew-derived strains, which needs to be further verified [45,46]. We also detected significant virulence differences among strains from different anatomical tissues of *C. chinensis*: strains from the head, digestive system, and female reproductive system had the highest virulence, while strains from the hindlegs, hindwings, and antennae were consistently less virulent. This variation is most likely driven by directional selection from the distinct microenvironments of different vector tissues: successful colonization of specific niches requires adaptive evolution of the pathogen, including upregulation of stress resistance, tissue adhesion, and immune evasion genes [47,48,49,50], which needs to be confirmed by further transcriptomic analysis.

The potential interaction between *E. amylovora* and *C. chinensis*—where the pathogen promotes vector reproduction [23] and the vector facilitates pathogen transmission and proliferation—creates a positive feedback loop that complicates pear fire blight management. Our findings highlight the need to develop integrated management strategies targeting both the vector and the pathogen simultaneously. Agents with dual insecticidal and bacteriostatic activity may effectively block this mutualistic cycle, and thus improve the efficiency of pear fire blight control in the field.

## 5. Conclusions

This study provides systematic evidence that *C. chinensis* serves as a competent vector of *E. amylovora*, the causal agent of pear fire blight, via both biological and mechanical transmission pathways. The pathogen was detected in 10 organs of adult psyllids, and strains from nymphs showed significantly higher pathogenicity than those from adults, which may be attributed to the feeding habits and immature immune system of nymphs. The nutrient-rich weakly acidic honeydew secreted by nymphs significantly promoted the proliferation and enhanced the virulence of *E. amylovora*. Phylogenetic analysis verified that all isolates shared high homology with typical *E. amylovora* strains. We further revealed a potential synergistic interaction mechanism: *C. chinensis* provides invasion wounds and nutrients for *E. amylovora*, while the pathogen may improve the fitness of its insect vector. As the first systematic report on the vector role of *C. chinensis* in *E. amylovora* transmission in Xinjiang pear orchards, this work fills a critical knowledge gap in the epidemic dynamics of pear fire blight in this region. These findings establish a theoretical basis for integrated pest–disease management and offer a key reference for developing dual-function insecticidal and bacteriostatic agents to block the transmission cycle.

## Data Availability

The sequences generated in this study are openly available in GenBank (NCBI) at the National Center for Biotechnology Information (NCBI) repository under the accession numbers (PV698613, PV698615, PV698614, PV698618, PV698617, PV698616, PX106820, PX609819, PX609820, PX609821, PX609822, PX609824, PX609825, PX609826, PX609823).

## Supplementary Materials

The following supporting information can be downloaded at: https://www.mdpi.com/article/10.3390/insects17050487/s1, Supplementary tables for pathogen identification, grading criteria for disease index and phylogenetic analysis. Table S1: Strains used in this experiment; Table S2: Grading criteria for disease index of isolated leaves; Table S3: Sequence information involved in systematic analysis.
