# Supplementary material for: Systematic Characterization of Cacopsylla chinensis as a Potential Vector of Erwinia amylovora on Korla Fragrant Pear in Xinjiang, China
Source: Insects. 2026 May 9;17(5):487. doi: 10.3390/insects17050487 (PMC13208058; doi:10.3390/insects17050487)
Supplement: Supplementary file 1 [file insects-17-00487-s001.zip › insects-4277519-supplementary.pdf]

Table S1. Strains used in this experiment

Number	Strains	Sources	Region	GenBank accession number
1	Y-1	*E. amylovora* isolated from Leaves of Korla fragrant pear damaged by *C. chinensis*	Xinjiang, China	PV698613
2	RN	within *C. chinensis* nymphs	Xinjiang, China	PV698615
3	RW	on the body surface of *C. chinensis* nymphs	Xinjiang, China	PV698614
4	CN	within adult *C. chinensis*	Xinjiang, China	PV698618
5	CW	on the body surface of adult *C. chinensis*	Xinjiang, China	PV698617
6	ML	Honeydew secreted by *C. chinensis* nymphs	Xinjiang, China	PV698616
7	PC	*E. amylovora* isolated from Korla Fragrant Pear Leaves	Xinjiang, China	PX106820
8	CJ	Antenna of adult *C. chinensis*	Xinjiang, China	PX609819
9	HC	Hindwings of adult *C. chinensis*	Xinjiang, China	PX609820
10	JC	Male reproductive system of adult *C. chinensis*	Xinjiang, China	PX609821
11	LC	Female reproductive system of adult *C. chinensis*	Xinjiang, China	PX609822
12	QC	Forewings of adult *C. chinensis*	Xinjiang, China	PX609824
13	TB	Head of adult *C. chinensis*	Xinjiang, China	PX609825
14	ZD	Midlegs of adult *C. chinensis*	Xinjiang, China	PX609826
15	QD	Forelegs of adult *C. chinensis*	Xinjiang, China	PX609823
16	HD	Hindlegs of adult *C. chinensis*	Xinjiang, China	Processing
17	XHD	Digestive system of adult *C. chinensis*	Xinjiang, China	Processing

Table S2. Grading criteria for disease index of isolated leaves

number of steps	Percentage of spots to total leaves
0	free of disease spots
I	Spot length 1% to 10% of inoculated leaves
III	Spot length 11%-20% of inoculated leaves
V	Spot length 21%~30% of inoculated leaves
VII	31%-45% of the length of the inoculated branch
IX	More than 45% of the length of the inoculated branch is covered by lesions

Table S3. Sequence information involved in systematic analysis

Strain number	16SrRNA GenBank Number	Country/Region
Eam 11-7	CP063688.1	America
Eam 32-10	CP063691.1	America
Eam Ea-102	CP104022.1	Xinjiang, China
Eam Ea-915	CP104025.1	Xinjiang, China
Pseudomonas syringae NZ_JBDFQP010000059.1	JBDFQP010000059.1	Canada
